# Death of Dr. James B. Rogers

**Published:** 1852-08

**Authors:** 


After we had gone to press with our last number, we learned the death of Dr. Jas. B. Rogers, Professor of Chemistry in the University of Pennsylvania.

Dr. Roger's health had been failing for some months past, from an attack of albuminuria, which proved fatal under a complication with pneumonia. He was elected professor of Chemistry in the University in 1847, while holding a similar position in Franklin Medical College. He was a popular and successful teacher in his department, and possessed in an eminent degree the love and confidence of all who knew him.

